# Effects of wrist extension on median nerve and flexor tendon excursions in patients with carpal tunnel syndrome: a case control study

**DOI:** 10.1186/s12891-021-04349-8

**Published:** 2021-05-24

**Authors:** Chien-Ting Liu, Dung-Huan Liu, Chii-Jen Chen, You-Wei Wang, Pao-Sheng Wu, Yi-Shiung Horng

**Affiliations:** 1 Department of Physical Medicine and Rehabilitation, Taipei Tzuchi Hospital, Buddhist Tzuchi Medical Foundation, No.289, Jianguo Rd., Xindian Dist., New Taipei City, 231 Taiwan; 2 Department of Medicine, Tzu Chi University, Hualien, Taiwan; 3 Department of Physical Medicine and Rehabilitation, China Medical University Hospital, Taichung, Taiwan; 4 Doctoral Degree Program of Biomedical Science and Engineering, College of Biological Science and Technology, National Chiao Tung University, Hsin-Chu, Taiwan; 5 Department of Medical Imaging and Radiological Technology, Yuanpei University of Medical Technology, Hsinchu, Taiwan; 6 Department of Computer Science and Information Engineering, National Taiwan University, Taipei, Taiwan

**Keywords:** Carpal tunnel syndrome, Median nerve, Flexor tendon, Doppler, Ultrasonography, Nerve gliding, Tendon gliding, Wrist extension

## Abstract

**Background:**

Reduced gliding ability of the median nerve in the carpal tunnel has been observed in patients with carpal tunnel syndrome (CTS). The purpose of this study was to evaluate the gliding abilities of the median nerve and flexor tendon in patients with CTS and healthy participants in the neutral and 30° extended positions of the wrist and to compare the gliding between the finger flexion and extension phases.

**Methods:**

Patients with CTS and healthy participants were consecutively recruited in a community hospital. All the subjects received the Boston CTS questionnaire, physical examinations, nerve conduction study (NCS), and ultrasonography of the upper extremities. Duplex Doppler ultrasonography was performed to evaluate the gliding abilities of the median nerve and flexor tendon when the subjects continuously moved their index finger in the neutral and 30° extension positions of the wrist.

**Results:**

Forty-nine patients with CTS and 48 healthy volunteers were consecutively recruited. Significant differences in the Boston CTS questionnaire, physical examination and NCS results and the cross-sectional area of the median nerve were found between the patients and the healthy controls. The degree of median nerve gliding and the ratio of median nerve excursion to flexor tendon excursion in the CTS group were significantly lower than those in the healthy control group in both the neutral and 30° wrist extension positions. Significantly increased excursion of both the median nerve and flexor tendon from the neutral to the extended positions were found in the CTS group. The ratio of median nerve excursion to flexor tendon excursion was significantly higher in the finger flexion phase than in the extended phase in both groups, and this ratio had mild to moderate correlations with answers on the Boston CTS Questionnaire and with the NCS results.

**Conclusions:**

Reduced excursion of the median nerve was found in the patients with CTS. The ratio of median nerve excursion to flexor tendon excursion was significantly lower in the patients with CTS than in the healthy volunteers. The median nerve excursion was increased while the wrist joint was extended to 30° in the patients with CTS. Wrist extension may be applied as part of the gliding exercise regimen for patients with CTS to improve median nerve mobilization.

**Supplementary Information:**

The online version contains supplementary material available at 10.1186/s12891-021-04349-8.

## Background

Carpal tunnel syndrome (CTS) is an entrapment neuropathy of the median nerve inside the carpal tunnel at the wrist level. The carpal tunnel contains the median nerve, nine flexor tendons, synovial bursae, and subsynovial connective tissue (SSCT). The median nerve and flexor tendons are normally connected by the multilayered SSCT [1]. Recent studies have revealed that fibrosis and increased thickness of the peritendinous SSCT of the flexor tendon aggravate adhesions inside the carpal tunnel and reduce gliding of the median nerve [2, 3]. The reported pathogenesis of CTS includes tenosynovitis of the flexor digitorum tendons, increased carpal tunnel pressure, and adhesion inside the carpal tunnel, which further interferes with the smooth gliding of the median nerve and induces repetitive trauma even under normal movement of the hand [4]. Moreover, extremely awkward wrist postures might also have adverse effects on the median nerve. For example, Loh and Muraki investigated the effect of wrist angles (neutral and 15°, 30°, and 45° flexion or extension) on the appearance of the median nerve at the proximal carpal tunnel; they found that the cross-sectional area (CSA) of the median nerve decreased as the wrist angle changed from neutral to flexion or extension. At 45° flexion or extension, the median nerve was smallest and most deformed [5].

Ultrasonography has been widely used in evaluating patients with CTS. In most studies, ultrasonography is used to diagnose CTS according to the following criteria: (1) increased CSA of the median nerve, (2) increased flattening ratio (FR; the ratio of the length of the long axis to that of the short axis of the median nerve), and (3) increased palmar bowing of the flexor retinaculum [6, 7]. Moreover, the swelling ratio (the ratio of the CSA of the median nerve at carpal tunnel to that at the forearm level), and ratio and difference in CSA between the median and ulnar nerves have also been used to accommodate intersubject variations [8, 9]. Some studies also observed increased intraneural blood flow in the median nerve on patients’ color or power Doppler ultrasonograms [10, 11].

In addition, several methods have been used in ultrasonography to estimate the gliding of the median nerve and flexor tendon. Some studies recorded the gliding of the median nerve and flexor tendon using B-mode ultrasonography and a cross-correlation algorithm to measure the longitudinal sliding frame by frame [12, 13]. They calculated the maximum correlation coefficient (*r*) for each pixel shift and determined the relative movement between the adjacent frames in sequences of images [12]. For example, Echigo et al. measured the median nerve gliding at the forearm and found that forearm supination is the preferred position for passive median nerve gliding exercise [14]. Korstanje et al. compared longitudinal excursion of the flexor tendons, the median nerve, and the SSCT between the more and less affected hands of patients with CTS during extension-to-fist motion. They found that excursions by the median nerve and the flexor digitorum superficialis (FDS) tendon were smaller in the more affected hands than in the less affected hands [3]. More recently, Bandaru et al. developed a singular value decomposition (SVD) filtering method, an improved correlation-based speckle tracking algorithm, to suppress the clutter in the background while tracking tendon motion [15]. They found that the relative displacement errors of the SVD filtering method were smaller than those of the original block-matching algorithm and of commercial tissue tracking software while using cadavers for ground truth displacement.

On the other hand, Hough et al. used duplex Doppler ultrasonography to evaluate the longitudinal excursion of the median nerve, and their results showed that patients with CTS had a smaller median nerve excursion than healthy controls in an elbow extended posture, but not in an elbow flexed posture [16–18]. They adjusted the Doppler sample volume indicator to lie within the median nerve and recorded the Doppler waveform while the subjects continuously moved their fingers. The area under the Doppler waveform in the velocity-time integral (VTI) spectrum represented the amount of median nerve excursion during each cycle of finger movements [17]. This method has been shown to have reliability, with an intraclass correlation coefficient of 0.92 [16]. Hough et al. also found that the ratio of median nerve excursion to flexor tendon excursion was significantly lower in patients with CTS than in controls when the elbow was extended and flexed [18]. Lopes et al. also used spectral Doppler ultrasonography to evaluate the median nerve and index FDS tendon excursions inside the carpal tunnel, and their results showed that healthy participants had greater median nerve excursions than symptomatic patients, and the ratio of median nerve excursion to FDS tendon excursion was lower in finger only motions, as compared to wrist motions with or without finger motion [19]. Ettema et al. also used a quantitative Doppler velocity analysis of eight cadaver wrists and found that the SSCT gliding velocities were statistically lower than the tendon velocities in the carpal tunnel [20].

Transverse plane ultrasonography has been used to evaluate the effects of finger flexion/extension motions on the displacement and deformation of the median nerves inside the carpal tunnel. For example, van Doesburg et al. found that the CSA and the perimeter of the median nerve were smaller in flexion than in extension with both index finger and thumb movements [21], whereas Toge et al. reported that active flexion of the thumb and individual fingers, particularly the index and middle fingers, produced significant transverse movement of the median nerve within the carpal tunnel without changing the CSA of the median nerve [22]. However, to the best of our knowledge, longitudinal median nerve excursion inside carpal tunnel in finger flexion and extension motion has not been compared extensively in patients with CTS. Although Yoshii et al. considered the speckle tracking method superior to the Doppler ultrasonographic method because of its potential usefulness for evaluating stress on the tendon and SSCT regardless of the angle between the object and the ultrasonographic transducer [23], duplex Doppler ultrasonographic method has the advantages of easily differentiating nerve and tendon gliding and defining the finger flexion and extension phases from the bidirectional Doppler waveforms. Therefore, we chose the duplex Doppler method to evaluate the gliding of the median nerve and flexor tendons in this study.

To date, no conclusive results have been reported regarding the gliding of the median nerve in patients with CTS. Some studies have shown reduced longitudinal gliding of the median nerve in patients with CTS [3, 18]; however, Erel et al. showed normal longitudinal sliding of the median nerve at the forearm level with a reduction in transverse nerve movement at the wrist level in patients with CTS [13]. Moreover, a previous study revealed that the combination of tendon gliding exercises with conventional treatments was more effective than that of nerve gliding exercises [24]. In fact, in that study, patients who were instructed to fully extend wrist joints during nerve gliding exercise exhibited mild deteriorations in functional status. This might have resulted from overstretching of the median nerve when the wrist is fully extended. Thus, in this study, we evaluated excursion of the median nerve and flexor tendon in the functional position of wrist joint (i.e., 30° extension). We hypothesized that 30° extension of the wrist is an appropriate position for nerve gliding exercise. Thus, the aims of this study were (1) to evaluate the excursion of the median nerve and flexor tendon in patients with CTS and healthy volunteers in the neutral and 30° extended positions of the wrist and (2) to compare the excursion of the median nerve and flexor tendon between active finger flexion and extension phases.

## Methods

### Patients and healthy participants

In this study, we recruited patients with CTS on a voluntary basis from the outpatient clinic of the department of physical medicine and rehabilitation at a community hospital. Healthy subjects were recruited from among the staff, patients’ families, and health volunteers in the hospital. Ethical approval to conduct the study was provided by our institutional review board, and informed consent was obtained from each participant. All methods in the study were carried out in accordance with the Helsinki guidelines and declaration. The inclusion criteria for the patient group included the presence of subjective symptoms (e.g., pain and/or numbness within the median nerve distribution of the digits) and either a positive Phalen or Tinel sign along with electrophysiological evidence of CTS. We excluded patients and healthy volunteers aged < 18 or > 65 years; those with underlying medical disorders such as diabetes mellitus, hypothyroidism, renal failure, or autoimmune disease; pregnant women; and people who had previous wrist trauma or surgery.

### Boston CTS questionnaire, physical examinations, and nerve conduction study

Each subject filled out the Boston CTS Questionnaire and underwent physical examinations, including testing for the Tinel and Phalen signs, pinch power, and Semmes-Weinstein monofilament sensory tests. The Boston CTS Questionnaire includes a symptom severity scale and a functional status scale. Scores for each scale range between 1 and 5; higher scores indicate greater disability [25]. To evaluate for the Tinel sign, the examiner tapped the median nerve along its course through the carpal tunnel, and the result was positive if the patient experienced tingling or numbness in at least one of the three radial digits [26]. To test for the Phalen sign, the participants were asked to fully flex both wrists for 60 s. A positive result was defined as the reproduction of the patient’s symptoms in the area innervated by the median nerve [26]. To perform the Semmes-Weinstein monofilament sensory test, a force-calibrated nylon filament was applied to seven sampling areas of the hand. If the participant could identify which digit was being tested with their eyes closed, the result was considered positive, and a weighted score from 1 to 5 was given to each filament according to the calculated score. The total score of the seven sampling areas was analyzed as a continuous variable [27].

A nerve conduction study (NCS) was performed using Neuropack M1 MEB-9200 J/K electrodiagnostic equipment (Nihon Kohden Corporation, Tokyo, Japan). While undergoing the NCS of the upper extremity, each participant was tested in the supine position in a quiet, air-conditioned room with the room temperature maintained at 26 °C. The NCS was performed using the supramaximal stimulation technique with constant current stimulator and surface recordings. The following criteria for the NCS of the median nerve were used to confirm the clinical diagnosis of CTS: (1) a distal motor latency > 4.4 ms, (2) a distal sensory latency > 3.4 ms, or (3) midpalm median nerve peak latency > 2.2 ms [28, 29].

### Ultrasonography

**Fig. 1:**
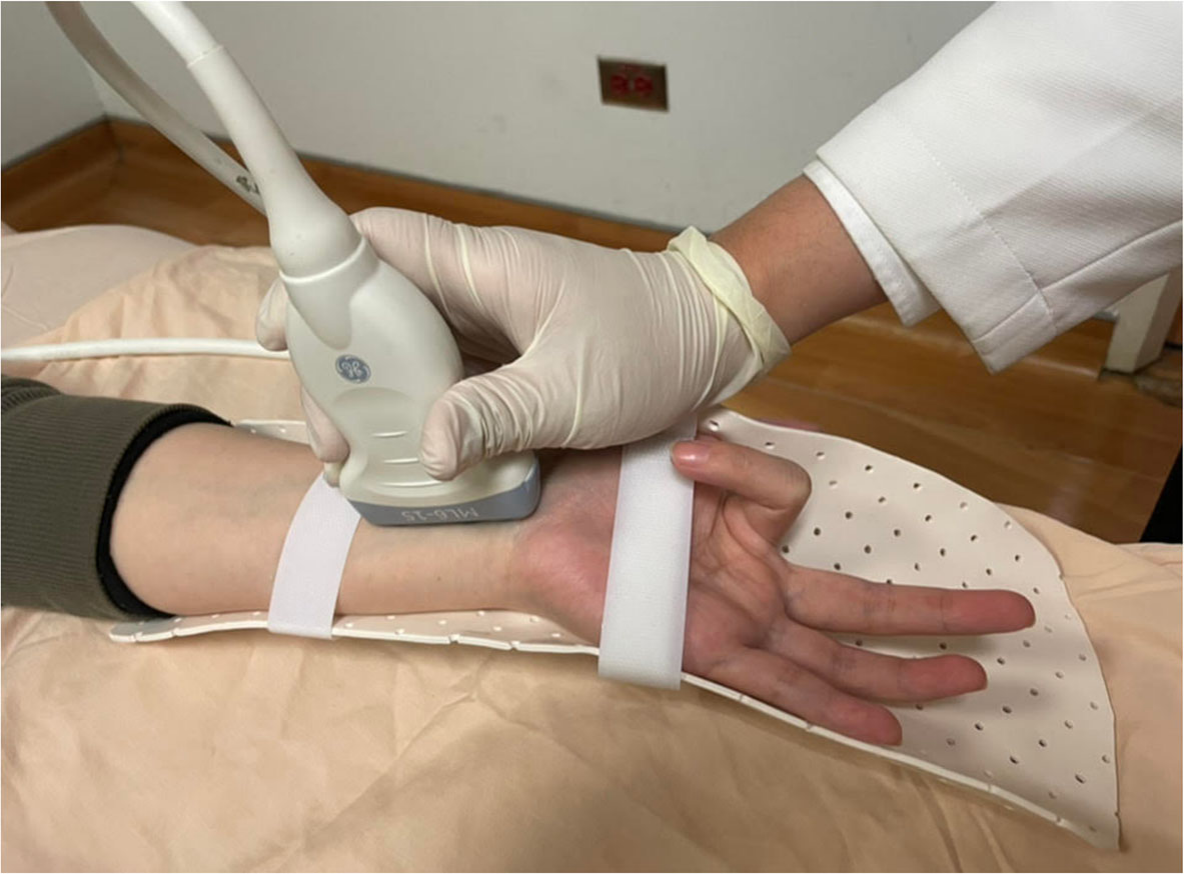
Study setup with the examinee’s hand on a splint with 30° extension and the ultrasound transducer longitudinally placed on the wrist

Ultrasonography was performed with a 12-MHz linear array transducer (GE LOGIQ 9; General Electric Medical Systems, Milwaukee, WI, USA). The participants were asked to lie in bed in the supine position, with their arms extended, forearms supinated, and wrists and hands resting on the bed. During ultrasonography, a splint was applied to keep the subject’s wrist in neutral and 30° extension positions, respectively (Fig. 1). The transducer was kept perpendicular to the surface of the median nerve to obtain the highest echogenic view. Pressure on the wrist was avoided during the entire scanning process to prevent further nerve deformation.

### Acquisition of transverse ultrasonography

**Fig. 2:**
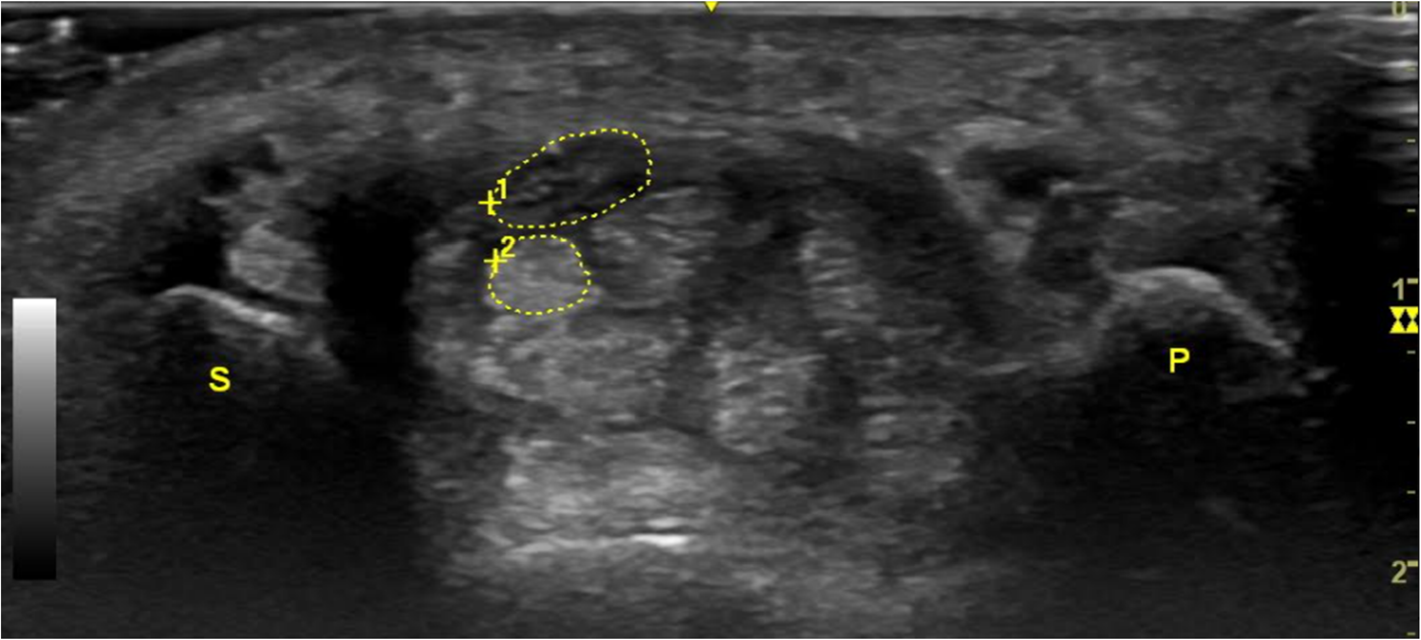
Cross sectional ultrasound image with outlined median nerve (upper encircled area) and flexor digitorum superficialis tendon of index finger (lower encircled area) at the pisiform level; P: pisiform bone; S: scaphoid bone

Transverse ultrasonograms were obtained at the pisiform level. Using electronic calipers, the CSA of the median nerve was measured by tracing the margin of the inner border of the perineural hyperechogenic rim that surrounded the hypoechoic median nerve (Fig. 2). We also calculated the FR by dividing the length of the long axis by that of the short axis of the median nerve at the pisiform level. All measurements were performed by one physiatrist who was board certified in ultrasonography with 6 years of experience in musculoskeletal ultrasonography and blinded to the clinical and NCS findings.

### Acquisition of duplex Doppler images

An ultrasonography probe was placed longitudinally at the level of the lunate–capitate intercarpal joint to obtain a clear image of the median nerve and FDS tendon. Longitudinal gliding excursions of the median nerve and flexor tendon were recorded using Doppler ultrasonography. A duplex Doppler mode (B/D mode) was used with a minimal sweep speed (*x*-axis, 5 s; movement, 1/s) and the lowest pulse repetition frequency (*y*-axis maximal velocity, 10 cm/sec). We adjusted the Doppler sample volume indicator to lie within the median nerve with a minimal sampling area (1 mm). The angle indicator line was also adjusted parallel to the median nerve. As suggested by Hough et al. [18], the beam-steering angle was set at 15° to reduce the beam-to-nerve angle to 45° to 60°. After the median nerve excursions were recorded, the sample volume indicator was then adjusted to lie within the FDS tendon at the same level, and the angle indicator line was adjusted parallel to the tendon. As the tendon excursion was larger than that of the median nerve, the pulse repetition frequency was increased to a *y*-axis maximal velocity of 40 cm/sec.

The participants were asked to move their index fingers repeatedly from full extension to semiflexion (90° flexion of the metacarpophalangeal and proximal interphalangeal joints). Before ultrasonography, participants were instructed to practice flexion of the metacarpal and proximal interphalangeal joints without flexing the distal interphalangeal joint to ensure that index digit motion was mainly caused by the FDS tendon. Moreover, during spectral Doppler measurement, participants were asked to look at their hand and ensure that metacarpal and proximal interphalangeal joints reached 90° flexion while continuously performing flexion and extension movement of the index finger. The index finger’s motion speed was controlled by a metronome, which maintained a frequency of 1 Hz for each cycle of motion (flexion and extension). Doppler images were recorded while the participants were alternatively performing flexion and extension of their index fingers in the neutral position and 30° extension of the wrist.

### Image analysis

**Fig. 3:**
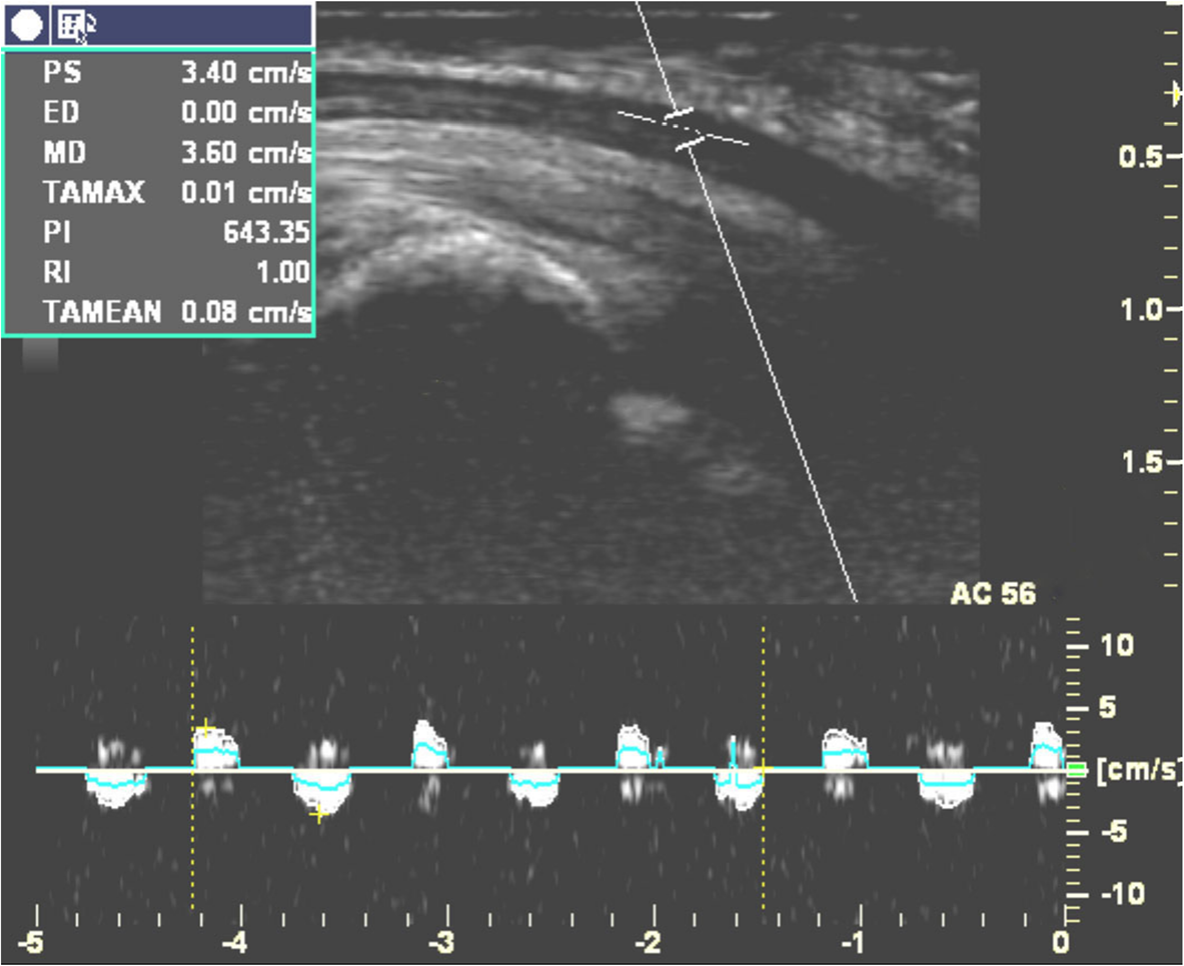
Doppler waveforms for median nerve excursion when subjects performed repetitive flexion/extension of index finger at a speed once per second

**Fig. 4:**
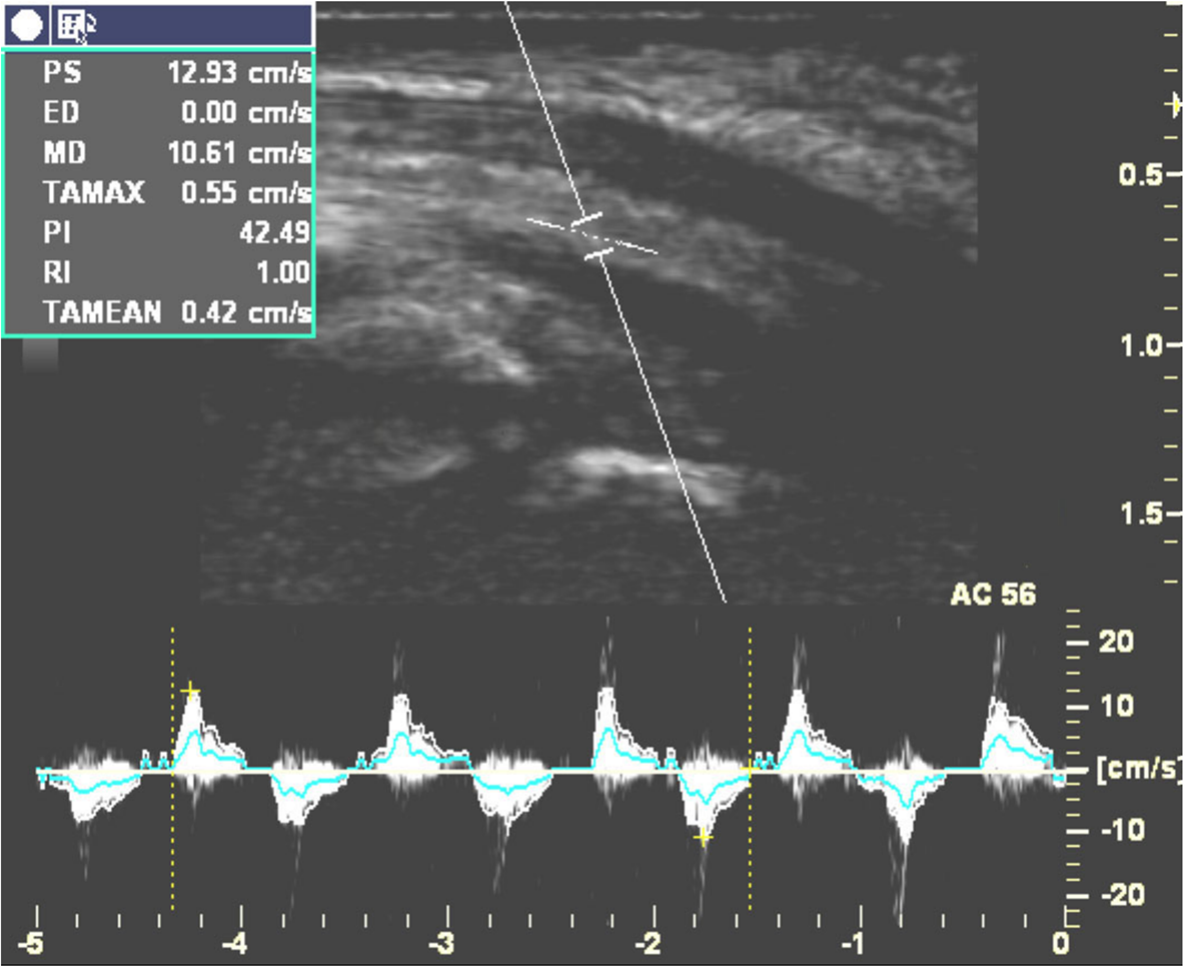
Doppler waveforms for tendon excursion of flexor digitorum superficialis when subjects performed repetitive flexion/extension of index finger at a speed once per second

Doppler waveforms representing the excursions of the median nerve or FDS tendon were saved. Each ultrasonogram contained at least three complete flexion/extension phases of the excursion waveforms (Figs. 3 and 4). The saved images were transferred to a personal computer and analyzed by a researcher who was unaware of the individual and group identity of each image. To obtain the total number of calibrated pixels within the area of a spike in the VTI of the Doppler spectrum, which represents the number of excursions of the median nerve or FDS tendon during each cycle of flexion/extension of the index finger, we used an in-house software developed by one of the authors (Additional file 1). To normalize the data, the ratio of the excursion of the median nerve to that of the FDS tendon was calculated in order to control for anthropometric variations between participants.

### Statistical analyses

The following data analyses were performed: (1) Student’s *t* and chi-square tests were used to compare the basic demographic data and answers on the Boston CTS Questionnaire between the patients and the controls. (2) To account for the correlation between both hands of the patients with bilateral CTS and the healthy controls, the generalized estimating equation was used to compare the results of physical examination, NCS, and B-mode ultrasonography between the hands with CTS and the healthy hands, after adjustment for age and sex. (3) The Wilcoxon signed-rank test was used for the Doppler ultrasonography data to compare the difference between the neutral and extended positions of the wrist in each group. This test was also used to evaluate the ratio of the excursion of the median nerve to that of the FDS tendon between the flexion and extension phases of finger motion, as these data were not normally distributed. (4) Spearman’s correlation was used to evaluate the relationship among the ratio of median nerve/FDS tendon excursion, the results of the Boston CTS questionnaire, and the results of the NCS. All of the statistical analyses were performed using SAS version 9.2 (SAS institute Inc., Cary, NC, USA).

## Results

Forty-nine patients with CTS who had either a positive Tinel or Phalen sign with positive NCS findings were consecutively recruited. Among the patients, 31 had bilateral CTS. Forty-seven age- and sex-matched healthy volunteers were recruited. As shown in Table 1, we found no significant differences in the demographic data between the patients and the healthy controls except in scores of the symptom severity scale and the functional status scale. We excluded 18 uninvolved hands and one hand with a bifid median nerve in the CTS group and 6 asymptomatic hands with abnormal NCS findings in the healthy group; therefore, 79 CTS hands and 88 healthy hands were included in the final analyses. Significant differences were found between the CTS and healthy groups in the physical examination results, NCS results, and CSA of the median nerve (Table 2).

**Table 1:** Frequency distribution of demographic data and CTS questionnaire for 49 patients with carpal tunnel syndrome (CTS) and 47 healthy volunteers

Characteristics	CTS patients N (%)	Healthy volunteers N (%)	***p*** value
**Personal Characteristics**			
Age, mean±SD, yrs	50.0±9.6	49.4±9.3	0.73
Female	44(89.8)	42(89.4)	0.94
Married	34(70.8)	31(68.9)	0.84
Employed	23(47.9)	32(68.1)	0.05
Smoking habit	1(2.1)	2(4.4)	0.61
Right-hand dominant	47(97.9)	43(97.7)	1.00
Bilateral hands involved	31(63.3)	-	-
**Educational Level**			0.10
College/University	17(35.4)	25(55.6)	
Senior High	21(43.8)	16(35.6)	
Junior High or below	10(20.8)	4(8.9)	
**Boston CTS questionnaire**			
symptom severity scale	2.6±0.7	1.2±0.4	<0.001
Functional status scale	1.7±0.6	1.1±0.1	<0.001

*P*-value, comparison between CTS patients and healthy volunteers by Student *t* or chi-square tests

**Table 2:** Comparison of clinical indicators of physical examination, nerve conduction studies, and B mode sonography between patients with carpal tunnel syndrome (CTS) and healthy volunteers

Variables	CTS hands (***n*** = 79)	Healthy hands (***n*** = 88)	***P*** value
	Mean±SD	Mean±SD	
Monofilament sensory test	30.1±4.1	32.8±3.2	0.0002
Pinch power (kg)	9.0±4.0	10.4±3.5	0.0854
Distal median sensory latency (ms)	3.8±1.3	2.6±0.4	<.0001
Distal median motor latency (ms)	5.0±1.1	3.4±0.4	<.0001
Midpalm median latency (ms)	2.3±0.5	1.5±0.2	<.0001
CSA of median nerve (mm^2^)	10.5±4.7	7.0±2.4	<.0001
Flattening ratio	2.8±0.8	2.6±0.9	0.1660

*P*-value, comparison between CTS patients and healthy volunteers after adjusting for age and gender (generalized estimating equation)

*CSA* cross-sectional area

We also found that the degree of median nerve excursion in the patients with CTS was significantly smaller than that in the healthy controls in both the neutral and extended positions of the wrist, but no significant difference in FDS tendon excursion was found between the two groups (Table 3). Significant improvements in the excursion of both the median nerve and flexor tendon from the neutral to the extended position were found in the CTS group. In the healthy group, only median nerve excursion significantly increased from the neutral to the extended positions. The ratio of median nerve excursion to FDS tendon excursion in the CTS group was also significantly smaller than that in the healthy group in both the neutral and extended positions of the wrist (Table 3). We further analyzed this ratio in the finger flexion and extension phases, and the results showed that the ratio of median nerve excursion to FDS tendon excursion was significantly smaller in the CTS group than in the healthy control group in both phases, either in the wrist neutral or extended positions (Table 4). Moreover, the ratio was significantly larger in the finger flexion phase than in the extension phase in both study groups. Table 5 also showed this ratio had mild to moderate correlations with answers on the Boston CTS Questionnaire (symptom severity and functional status scales) and with the NCS results (distal median motor and sensory latency and midpalm latency).

**Table 3:** Comparison of gliding of the median nerve and flexor digitorium superficialis tendon between patients with carpal tunnel syndrome (CTS) and healthy volunteers, while performing flexion and extension movements of index finger at a speed of one cycle per second

Variables	CTS hands (***n*** = 79)	Healthy hands (***n*** = 88)	***p***- value^**b**^
	Mean±SD	Mean±SD	
**Median nerve excursion (mm)**			
Wrist in neutral position	18.5±7.0	23.7±9.1	0.0001
Wrist in extended position	21.3±10.6	25.6±10.3	0.02
*p*- value^a^	0.004	0.017	
**Flexor tendon excursion (mm)**			
Wrist in neutral position	99.1±30.7	89.7±29.1	0.11
Wrist in extended position	109.0±34.2	96.4±30.8	0.05
*p*- value^a^	0.047	0.213	
**Ratio of median nerve/flexor tendon excursion**			
Wrist in neutral position	0.20±0.11	0.29±0.15	0.0008
Wrist in extended position	0.21±0.11	0.29±0.14	0.005
*p*- value^a^	0.728	0.909	

^a^comparison between wrist at neutral and extended positions (Wilcoxon Signed Ranks test)

^b^comparison between CTS patients and healthy volunteers after adjusting for age and gender (generalized estimating equation)

**Table 4:** Comparison of the ratio of median nerve excursion to flexor tendon excursion between finger flexion and extension phases in patients with carpal tunnel syndrome (CTS) and healthy volunteer, while performing flexion and extension movements of index finger at a speed of one cycle per second

Variables	CTS hands (***n*** = 79)	Healthy hands (***n*** = 88)	***p***- value^**b**^
	Mean±SD	Mean±SD	
**Ratio of median nerve/flexor tendon excursion in wrist neutral position**			
Flexion phase	0.22±0.11	0.30±0.16	0.0009
Extension phase	0.18±0.10	0.27±0.15	0.0004
*p*- value^a^	0.001	0.001	
**Ratio of median nerve/flexor tendon excursion in wrist extended position**			
Flexion phase	0.23±0.14	0.31±0.16	0.0029
Extension phase	0.20±0.12	0.27±0.14	0.0024
*p*- value^a^	0.007	0.004	

^a^comparison between wrist at neutral and extended positions (Wilcoxon Signed Ranks test)

^b^comparison between CTS patients and healthy volunteers after adjusting for age and gender (generalized estimating equation)

**Table 5:** Correlation matrix among the ratio of median nerve/flexor digitorum superficialis tendon excursion at neutral position, the results of the Boston carpal tunnel syndrome questionnaire and nerve conduction study of the median nerve

	Ratio of median nerve/flexor tendon excursion	Symptom severity scale	Functional status scale	Midpalm latency	Distal sensory latency	Distal motor latency
**Ratio of median nerve/flexor tendon excursion**	1.00					
**Symptom severity scale**	-0.33^a^	1.00				
**Functional status scale**	-0.31^a^	0.78^a^	1.00			
**Midpalm latency**	-0.32^a^	0.68^a^	0.53^a^	1.00		
**Distal sensory latency**	-0.36^a^	0.65^a^	0.54^a^	0.79^a^	1.00	
**Distal motor latency**	-0.31^a^	0.72^a^	0.63^a^	0.75^a^	0.83^a^	1.00

^a^Correlation is significant at the 0.01 level (two-tailed)

## Discussion

In this study, we observed not only decreased excursion of the median nerve but also a decreased ratio of median nerve excursion to FDS tendon excursion in the patients with CTS, in comparison with healthy controls. Because FDS tendon excursion did not differ significantly between the patients with CTS and the healthy controls, decreased excursion of the median nerve resulted in decreased median nerve/FDS tendon excursion ratio in patients with CTS. This implies that fibrosis and adhesions in the carpal tunnel hinders excursion of the median nerve but not of the FDS tendon in CTS. Although we evaluated different digits, our results were similar to those of Filius et al., who found smaller median nerve displacement in patients with CTS and no difference in flexor tendons displacement of the middle finger between the patients and the healthy controls [30]. Moreover, they demonstrated that the ratio of the median nerve excursion to FDS tendon excursion tended to decrease when severity of the CTS increased [30]. Our results also showed that this ratio was weakly to moderately correlated with clinical symptoms (symptom severity and functional status scores) and NCS results (distal median motor and sensory latency and midpalm latency; Table 5). We recommend further randomized controlled trials to evaluate the effects of nerve gliding exercise on the clinical indicators and the ratio of the median nerve/flexor tendon excursion.

In the patients with CTS, we also found increases in median nerve and FDS tendon excursions when the wrist joint was extended 30° from the neutral position. As shown in a previous study by Nanno et al. wherein transverse plane ultrasonography was used, the median nerve moved significantly more dorsally during finger motion when the wrist was in extension [31]. This might imply that the median nerve moves away from the transverse carpal ligament when the wrist is extended dorsally, and this commonly used wrist extension movement in nerve gliding exercise improved the mobility of the median nerve in the patients with CTS.

On the contrary, the wrist flexion position might reduce the carpal tunnel space and increase the shear strain in the SSCT. A previous cadaver study found that shear strain in the median nerve was greatest in the 60° wrist flexion posture [32]. Longitudinal excursion of the median nerve inside the carpal tunnel may also be influenced by the position of other joints in the upper extremity. Although the study of Dilly et al. showed an additional strain of 2.5 to 3.0% of the median nerve excursion in the forearm when the arm was at 90° shoulder abduction and 60° wrist extension, and the elbow was straight in healthy subjects [33]; our study revealed significant increases in the median nerve and flexor tendon excursions with a 30° wrist extension in the patients with CTS. A previous study also showed the relative motion between the FDS tendon and SSCT decreased in 30° wrist extension [34]. Thus, 30° wrist extension may stretch fibrous adhesions inside the carpal tunnel without overstraining the median nerve and flexor tendon in patients with CTS. However, further degree of wrist extension should be avoided because it might induce the tendons of the flexor digitorum superficialis and profundus muscles to migrate into the proximal inlet of the carpal tunnel, which reduces the space inside the carpal tunnel and compresses the median nerve [4].

We also found greater flexor tendon excursion in the patients with CTS than in the healthy volunteers, especially in the wrist extension position, although the difference was only borderline significant (*p* = 0.05; Table 3). During finger motion, the flexor tendons moved actively, and their movements were associated with both the synovium- and paratenon-related sources of friction [35], whereas the median nerve moved less than the flexor tendon and was passively glided by indirect traction from the flexor tendons via SSCT [1]. While performing continuous flexion or extension movements of the index finger at a fixed speed, patients with CTS might need to make more effort and have greater flexor tendon excursion than healthy subjects to overcome the fibrous adhesion inside the carpal tunnel, especially when the median nerve and flexor tendons were stretched in wrist extended position. As a result, the ratio of median nerve excursion to flexor tendon excursion was much smaller in the patients with CTS than in the healthy volunteers (Table 3), which collaborated previous literature [18].

Moreover, in both study groups, the ratio of median nerve excursion to FDS tendon excursion was significantly larger in the flexion phase of the index motion than in the extension phase, either in the wrist neutral or extended position (Table 4). The possible explanation is that both the median nerve and FDS tendon were mildly stretched when the finger extended to 0°, in comparison with resting position; therefore, when the index finger performed flexion motion from an extended position, excursion of the FDS tendon might increase, and this increase might induce more passive displacement of the median nerve. Previous ultrasonographic studies in the transverse plane also demonstrated that active finger flexion induced passive displacement of the median nerve into the ulnar direction and then back to the dorsal or radial direction when the finger moved from the flexion to the extension position [31, 36]. Therefore, friction between the median nerve and the flexor tendons may increase and slow down the median nerve excursion in the finger extension phase. As suggested by Coppieters et al., exercise program was recommended to address the alternative wrist extension/finger flexion and wrist flexion/finger extension movements to minimize median nerve strain [37].

This study had the following limitations: (1) Spectral broadening is an important artifact in pulsed wave Doppler ultrasonographic imaging, so the results might overestimate the tendon and nerve excursions if the intrinsic spectral broadening was not accurately corrected [18]. Thus, we corrected this factor accordingly and calculated the ratio of nerve excursion to tendon excursion. This ratio can also control for intersubject anthropometric variations. (2) Doppler imaging is restricted to longitudinal movement and is limited by transverse movement beyond the beam width [12]; thus we could not capture out-of-plane movements. (3) Owing to background noise, the small excursion of the median nerve is more difficult to record than the flexor tendon excursion on Doppler ultrasonography. To overcome this issue, we lowered the gain and repetition pulsed frequency and asked the subjects to continuously flex/extend their index fingers until three consecutive clear waveforms of nerve excursion were obtained. (4) Because spectral Doppler is unable to evaluate the nerve and tendon excursion from the same motion cycle, we used a metronome to control the speed of finger motion and first measured the median nerve excursion and then the FDS tendon excursion while patients were continuously flexing and extending their index finger in rhythm with the metronome at 1 Hz.

## Conclusions

Reduced median nerve excursion was found in the patients with CTS. The ratio of median nerve excursion to flexor tendon excursion was significantly smaller in the patients with CTS than in the healthy volunteers. Median nerve excursion was increased while the wrist joint was extended to 30° in the patients with CTS. Wrist extension may be applied as part of the gliding exercise regimen for patients with CTS to improve median nerve mobilization. Further randomized controlled trials are recommended for evaluating the effects of gliding exercise on the clinical indicators and the ratio of the median nerve/flexor tendon excursion.

## Supplementary Information

**Additional file 1.**

## Data Availability

All the data needed to achieve the conclusion are contained within the paper. The raw data cannot be shared publicly due to ethical reason.

## Abbreviations

CSA: Cross-sectional area
CTS: Carpal tunnel syndrome
FDS: Flexor digitorum superficialis
FR: Flattening ratio
NCS: Nerve conduction study
SSCT: Synovial bursae, and subsynovial connective tissue
SVD: Singular value decomposition
VTI: Velocity-time integral
