# Supplementary material for: Effects of wrist extension on median nerve and flexor tendon excursions in patients with carpal tunnel syndrome: a case control study
Source: BMC Musculoskelet Disord. 2021 May 24;22:477. doi: 10.1186/s12891-021-04349-8 (PMC8146623; doi:10.1186/s12891-021-04349-8)
Supplement: Supplementary file 1 — Additional file 1. [file 12891_2021_4349_MOESM1_ESM.docx]

1. **Doppler Area Calculation**

Herein we used ultrasonic images to segment the Doppler area for evaluating corresponding variations between the median nerve and tendon. We proposed a powerful segmentation framework to obtain and calculate the Doppler area. The proposed segmentation framework included four procedures: region of interest (ROI) selection, image preprocessing, image segmentation, and area calculation, as shown in Figure 1.

Figure 1. Flowchart of the proposed segmentation framework.

- 1. ***ROI Selection and Image Preprocessing***

ROI was selected in the original ultrasonic image (red region in Figure 2). However, imaging speckle noise is a common phenomenon in ultrasonic pulse-echo measurements. In general, speckle is a type of noise that needs to be reduced using image processing techniques (1, 2). Before image segmentation, image preprocessing with median filtering was adopted to reduce the speckle noise in the ultrasonic images.

Median filtering is a smooth filtering. Although all smoothing methods are effective in eliminating noise, they adversely affect the edges in the image. Therefore, binary threshold was used for enhancing the edge to separate the foreground and background in the ROI. Otsu’s algorithm (1, 2) was used to apply the binary threshold procedure; using this algorithm, the obvious edges could be preserved and could be the superior features in the image. The threshold value of the binary procedure was assigned 120 of the intensity value using Otsu’s algorithm, as shown in Figure 3. The binary threshold result using Otsu’s algorithm is shown in Figure 4.


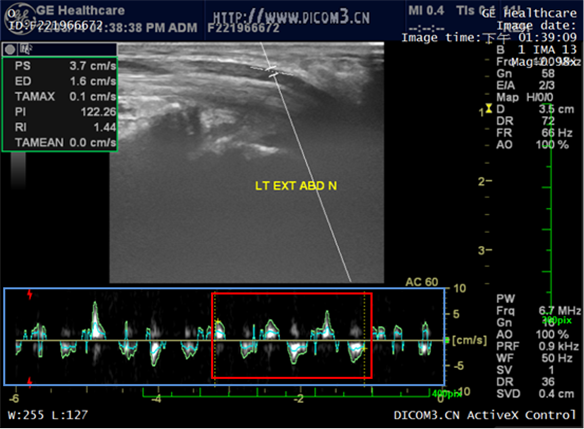


Figure 2. Region of interest (red region) in the original ultrasonic image.


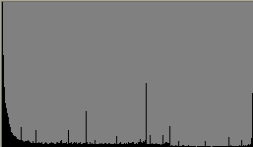


Figure 3. An example of a threshold value for Otsu’s algorithm.


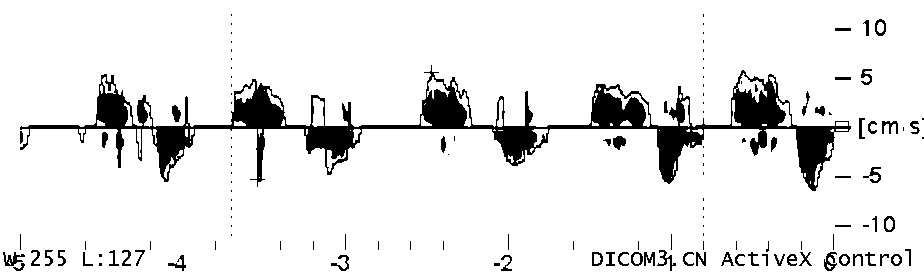


Figure 4. Result of the image revised for the binary threshold image.

- 1. ***Image Segmentation***

Following image preprocessing, the region growing algorithm (3, 4) was used to segment the Doppler area. Region growing is a region-based image segmentation method that enables setting an initial seed point and then determines whether all pixel neighbors should be marked to the same region with the seed point. Herein the traditional region growing method was modified. First, we set more than one seed points. Second, we changed the recursive growing rule and added a dynamic programming method into the segmentation procedure. In the modified region growing method, we sped up the growing time and obtained more precise segmentation results. The red region was grown using the modified region growing method, as shown in Figure 5.

After the image segmentation procedure, the segmented and original images were merged with the proposed segmentation system. Examples of Doppler area segmentation are shown in Figure 6.


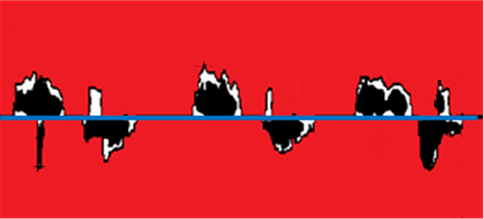


Figure 5. The red region is grown using the modified region growing method.


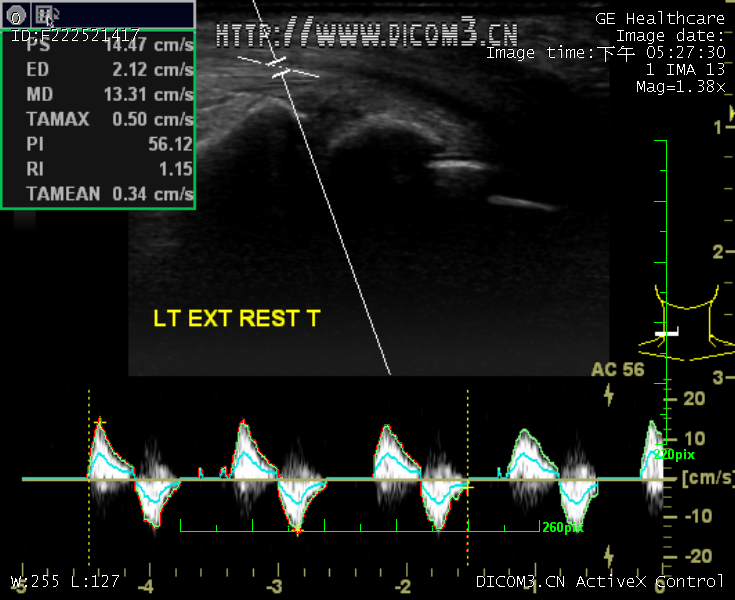

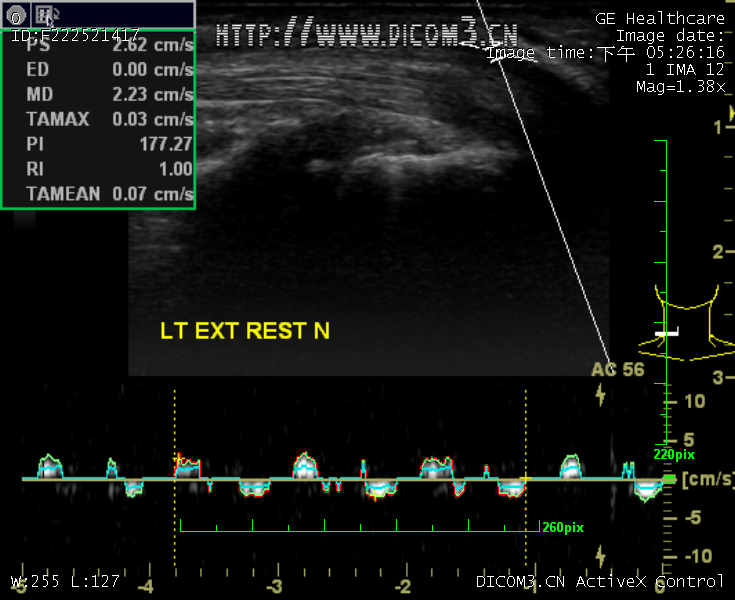


Figure 6. Two merged results of Doppler area segmentation using the modified region growing method.

- 1. ***Area Calculation***

For calculation of the Doppler area, the area of the non-red region in Figure 5 was calculated, as defined in Eqn. (1).

 (1)

where *A_Dopp_* is the area of Doppler wave; *A_ROI_* is the area of the whole ROI; *A_R_* is the area of the red region generated using the modified region growing method. However, as shown in Figure 2, the unit of measurement for the Doppler wave area was millimeter, the abscissa represented the number of periods spanned by the time variable (unit: seconds), and the ordinate represented the scaling factor as amplitude (unit: mm/sec). Therefore, we defined the Doppler wave area as distance (5). Given the distance of the Doppler wave $D_{W}$*D_w_*, we used the following equation to calculate the distance.

 (2)

where angle *θ* is the Doppler angle. When Eqn. (1) and (2) were combined, the final area (or distance) of the Doppler wave was as follows:

 (3)

***(4) Reliability and Validity***

Regarding the accuracy of the measurements, the results of our proposed software were tested by radiologists and medical image processing experts. This method presents a semi-automatic processing procedure. We tested all cases through intersection over union (IoU), which calculates how much area was segmented without involving unnecessary regions, using the following equation:

$IoU=\frac{RESULT\cap GT}{RESULT\cup GT}$,

where *RESULT* is the output of the proposed software and *GT* is the ground truth that is manually generated by an expert. The value of IoU was over than 0.85 for all the tested cases. The result shows that the proposed software has high reliability and validity and is operator independent.

**References**

1. Chen CJ, Chang RF, Moon WK, Chen DR, Wu HK. 2-D ultrasound strain images for breast cancer diagnosis using nonrigid subregion registration. Ultrasound in medicine & biology. 2006;32(6):837-46.

2. Moon WK, Chang RF, Chen CJ, Chen DR, Chen WL. Solid breast masses: classification with computer-aided analysis of continuous US images obtained with probe compression. Radiology. 2005;236(2):458-64.

3. Chen CJ, Wang YW. A Preoperative 3D Computer-Aided Segmentation and Reconstruction System for Lung Tumor. Journal of Communication and Computer. Apr. 2012;9(4):422-5.

4. Wang YW, Chen CJ. An Efficient Segmentation Framework for Continuous CT Images. Journal of Image Processing and Communication. Dec. 2010;2(1):31-4.

5. Hough AD, Moore AP, Jones MP. Reduced longitudinal excursion of the median nerve in carpal tunnel syndrome. Archives of physical medicine and rehabilitation. 2007;88(5):569-76.
